# Supplementary material for: Association between blood lipid levels and risk of gastric cancer: A systematic review and meta-analysis
Source: PLoS One. 2023 Jul 7;18(7):e0288111. doi: 10.1371/journal.pone.0288111 (PMC10328306; doi:10.1371/journal.pone.0288111)
Supplement: S1 Table — (PDF) [file pone.0288111.s002.pdf]

S1 Table. Searching strategy for PubMed.

#1	"Stomach Neoplasms"[Mesh]
#2	“Neoplasm, Stomach”[Title/Abstract] OR “Stomach Neoplasm”[Title/Abstract] OR “Neoplasms, Stomach”[Title/Abstract] OR “Gastric Neoplasms”[Title/Abstract] OR “Gastric Neoplasm”[Title/Abstract] OR “Neoplasm, Gastric”[Title/Abstract] OR “Neoplasms, Gastric”[Title/Abstract] OR “Cancer of Stomach”[Title/Abstract] OR “Stomach Cancers”[Title/Abstract] OR “Gastric Cancer”[Title/Abstract] OR “Cancer, Gastric”[Title/Abstract] OR “Cancers, Gastric”[Title/Abstract] OR “Gastric Cancers”[Title/Abstract] OR “Stomach Cancer”[Title/Abstract] OR “Cancer, Stomach”[Title/Abstract] OR “Cancers, Stomach”[Title/Abstract] OR “Cancer of the Stomach”[Title/Abstract] OR “Gastric Cancer, Familial Diffuse”[Title/Abstract]
#3＝#1 OR #2	
#4	“Lipids/blood"[Mesh] OR "Triglycerides"[Mesh] OR "Cholesterol"[Mesh]
#5	"Triacylglycerol"[Title/Abstract] OR "Triacylglycerols"[Title/Abstract] OR "Epicholesterol"[Title/Abstract] OR "alpha Lipoprotein Cholesterol"[Title/Abstract] OR "Cholesterol alpha Lipoprotein"[Title/Abstract] OR "HDL Cholesterol"[Title/Abstract] OR "High Density Lipoprotein Cholesterol"[Title/Abstract] OR "Cholesterol HDL2"[Title/Abstract] OR "HDL2 Cholesterol"[Title/Abstract] OR "Cholesterol HDL3"[Title/Abstract] OR "HDL3 Cholesterol"[Title/Abstract] OR " Low Density Lipoprotein Cholesterol "[Title/Abstract] OR "beta Lipoprotein Cholesterol"[Title/Abstract] OR "Cholesterol beta Lipoprotein"[Title/Abstract] OR "LDL Cholesterol"[Title/Abstract] OR "Cholesteryl Linoleate LDL"[Title/Abstract] OR "LDL Cholesteryl Linoleate"[Title/Abstract] OR "lipids"[Title/Abstract] OR "serum lipid"[Title/Abstract] OR "Triglycerides"[Title/Abstract] OR "Triglyceride"[Title/Abstract] OR "Cholesterol"[Title/Abstract] OR "Total Cholesterol"[Title/Abstract] OR "TG"[Title/Abstract] OR "TC"[Title/Abstract] OR "HDLC"[Title/Abstract] OR "LDL-C"[Title/Abstract]
#6＝#4 OR #5	
#7＝#3 AND #6	
