# Supplementary material for: Association between blood lipid levels and risk of gastric cancer: A systematic review and meta-analysis
Source: PLoS One. 2023 Jul 7;18(7):e0288111. doi: 10.1371/journal.pone.0288111 (PMC10328306; doi:10.1371/journal.pone.0288111)
Supplement: S2 Table — (PDF) [file pone.0288111.s003.pdf]

**S2 Table. Characteristics of the included studies.**

| First author<br>Publication year<br>Country<br>(Cohort studies) | Case/subject<br>Duration of follow-up<br>(years) | Exposure categories | HR/RR (95%CI) | Adjustment for potential confounding factors | NOS |
|---|---|---|---|---|---|
| Lim 2022<br>Korea [7]. | 17649/2,722,614<br>8.26 years | Total:<br>TC ≥ 224 vs < 175 mg/dL<br>HDL ≥ 66 vs < 48mg/dL<br>LDL ≥ 141 vs < 96mg/dL<br>TG ≥ 145 vs < 71mg/dL | <br>0.92 (0.88,0.96)<br>0.90 (0.87,0.94)<br>0.96 (0.92,1.01)<br>0.99 (0.95,1.04) | Age, cigarette smoking, alcohol consumption, regular exercise, BMI, diabetes mellitus, cholesterol-lowering agent | 7 |
| Iso 2009<br>Japan [20] | 557/33,368<br>12.4 years | Man:<br>TC 6.21≥ vs <4.14mmol/L<br>Woman:<br>TC 6.21≥ vs <4.14mmol/L | <br>0.81 (0.73,0.91)<br><br>0.97 (0.84,1.12) | Age, BMI, pack year of smoking, ethanol intake, hypertension, diabetes, hyperlipidemia medication use, total vegetable intake, coffee intake | 8 |
| Guan 2018<br>China [15] | 148/68,759<br>8 years | Total:<br>TC ≥5.60 vs <4.31mmol/L<br>LDL ≥2.86 vs <1.89mmol/L | <br>0.63 (0.31,1.27)<br>0.59 (0.26,1.31) | Age, cigarette smoking, alcohol consumption, physical activity, hypertension, diabetes, BMI | 8 |
| Tornberg 1988<br>Switzerland [18] | 576/92,710<br>20 years | Total:<br>TC >294 vs <220 mg/dL<br>Man:<br>TC >294 vs <220 mg/dL<br>Woman:<br>TC >294 vs <220 mg/dL | <br>0.85 (0.7,0.93)<br><br>0.87 (0.78,0.97)<br><br>0.83 (0.73,0.94) | Age, gender, region | 7 |
| Kitahara 2011<br>Korea [23] | 18,012/756,604<br>14 years | Man:<br>TC≥ 240 vs < 160 mg/dL<br>Woman:<br>TC ≥ 240 vs < 160 mg/dL | <br>0.87 (0.82 ,0.93)<br><br>0.86 (0.77,0.97) | Cigarette smoking, alcohol drinking, body mass index, fasting serum glucose, hypertension, physical activity | 7 |
| Ahn 2009<br>Finland [16] | 334/29093<br>14.9 years | Total:<br>TC ≥276.7 vs <203.9 mg/dL<br>HDL ≥55.3 vs <36.2 mg/dL | <br>0.86 (0.62,1.21)<br>0.9 (0.61,1.32) | Age, intervention, level of education, blood pressure, BMI, physical activity, smoking, saturates fat intake, polyunsaturated fat intake, total calorie, alcohol consumption, serum HDL cholesterol. | 8 |

| | | | | | |
|---|---|---|---|---|---|
| Asano 2007<br>Japan [19] | 2604/97 2007<br>14 years | Total： | | Age, sex, H. pylori infection, atrophic gastritis, family history of malignant neoplasm, smoking status, body mass index, hemoglobin A1c, white blood cell count, and intake of total energy, salt, vitamin A and vitamin B1 | 8 |
| | | TC ≥6.05 vs <4.60 mmol/L | 0.81（0.63,0.97） | | |
| | | Man： | | | |
| | | TC ≥5.77 vs <4.42mmol/L | 0.78（0.60,1.01） | | |
| | | Woman： | | | |
| | | TC≥6.26 vs <4.78mmol/L | 0.83（0..63,1.25） | | |
| Lin 2015<br>Norway [21] | 437/192,903<br>10.6 years | Total： | | Age, gender, BMI, education, smoking, family history | 7 |
| | | TG ≥1.7 vs <1.7 mmol/L | 1.02 (0.83,1.26) | | |
| | | HDL ≥1 vs <1mmol/L | 0.85（0.66,1.08） | | |
| | | Man： | | | |
| | | TG ≥1.7 vs <1.7 mmol/L | 1 (0.67,1.49) | | |
| | | HDL ≥1 vs <1mmol/L | 0.98 (0.64,1.49) | | |
| | | Woman： | | | |
| | | TG ≥1.7 vs <1.7 mmol/L | 0.95 (0.72,1.25) | | |
| | | HDL ≥1.3 vs <1.3 mmol/L | 0.75 (0.53,1.03) | | |
| Ulmer 2009<br>Austria [24] | 315/156 153<br>15 years | Total： | | BMI, smoking | 8 |
| | | TG：Q4 vs Q1 | 1.45 (0.97,2.17) | | |
| Wulaningsih 2012<br>Sweden [17] | 776/ 540309<br>12 years | Total： | | Age, gender, fasting status, glucose | 8 |
| | | TC ＜4.7 vs ≥6.30mmol/L | 1.03 (0.80–1.32) | | |
| | | TG ＜0.7 vs ≥1.60mmol/L | 1.17 (0.90–1.55) | | |
| | | HDL ＜1.25 vs ≥1.08mmol/L | 0.62 (0.33–1.17) | | |
| | | LDL ＜2.82 vs ≥4.25mmol/L | 0.80 (0.30–2.08) | | |

HR: Hazard Ratio, RR: Relative Ratio, BMI: Body Mass Index.
