# Supplementary material for: Association between blood lipid levels and risk of gastric cancer: A systematic review and meta-analysis
Source: PLoS One. 2023 Jul 7;18(7):e0288111. doi: 10.1371/journal.pone.0288111 (PMC10328306; doi:10.1371/journal.pone.0288111)
Supplement: Attachment — Submitted filename: Response to Reviewers.docx [file pone.0288111.s005.docx]

Dear Academic Editor and Reviewers:

Thank you for your feedback on our manuscript. We have carefully reviewed your comments and suggestions and made the necessary revisions accordingly. Please find our responses to each of your comments listed below:

**Response to Academic Editor:**

1.Please insert quality assessment score in supplementary Table-S2.

-Update PRISMA flowchart in accordance with PRISMA 2020

Response: Thank you for your feedback regarding our manuscript. We have carefully considered your suggestions and made the necessary revisions.

To incorporate the quality assessment of the literature, we have merged it into the original Table S2 and generated a new document, which we have renamed as S2 Table.

Additionally, we have updated the PRISMA flowchart in accordance with the PRISMA 2020 guidelines as per your recommendation.

Thank you again for your valuable input, and please let us know if you have any further suggestions or comments.

2. Please ensure that your manuscript meets PLOS ONE's style requirements, including those for file naming. The PLOS ONE style templates can be found at

https://journals.plos.org/plosone/s/file?id=wjVg/PLOSOne_formatting_sample_main_body.pdf and

<https://journals.plos.org/plosone/s/file?id=ba62/PLOSOne_formatting_sample_title_authors_affiliations.pdf>

Response: Thank you for your feedback. We apologize for any inconvenience caused by not adhering to PLOS ONE's style requirements, including those for file naming. We have revised our manuscript to ensure that it meets the specified guidelines. If you have any further suggestions or comments, please let us know. We appreciate your time and attention to our submission.

3.In your Data Availability statement, you have not specified where the minimal data set underlying the results described in your manuscript can be found. PLOS defines a study's minimal data set as the underlying data used to reach the conclusions drawn in the manuscript and any additional data required to replicate the reported study findings in their entirety. All PLOS journals require that the minimal data set be made fully available. For more information about our data policy, please see <http://journals.plos.org/plosone/s/data-availability>.

Upon re-submitting your revised manuscript, please upload your study’s minimal underlying data set as either Supporting Information files or to a stable, public repository and include the relevant URLs, DOIs, or accession numbers within your revised cover letter. For a list of acceptable repositories, please see http://journals.plos.org/plosone/s/data-availability#loc-recommended-repositories. Any potentially identifying patient information must be fully anonymized.

Important: If there are ethical or legal restrictions to sharing your data publicly, please explain these restrictions in detail. Please see our guidelines for more information on what we consider unacceptable restrictions to publicly sharing data: http://journals.plos.org/plosone/s/data-availability#loc-unacceptable-data-access-restrictions. Note that it is not acceptable for the authors to be the sole named individuals responsible for ensuring data access.

We will update your Data Availability statement to reflect the information you provide in your cover letter.

Upon re-submitting your revised manuscript, please upload your study’s minimal underlying data set as either Supporting Information files or to a stable, public repository and include the relevant URLs, DOIs, or accession numbers within your revised cover letter. For a list of acceptable repositories, please see http://journals.plos.org/plosone/s/data-availability#loc-recommended-repositories. Any potentially identifying patient information must be fully anonymized.

Important: If there are ethical or legal restrictions to sharing your data publicly, please explain these restrictions in detail. Please see our guidelines for more information on what we consider unacceptable restrictions to publicly sharing data: http://journals.plos.org/plosone/s/data-availability#loc-unacceptable-data-access-restrictions. Note that it is not acceptable for the authors to be the sole named individuals responsible for ensuring data access.

We will update your Data Availability statement to reflect the information you provide in your cover letter.

Response: Thank you for your feedback on our manuscript. We appreciate the opportunity to clarify our data availability statement. We understand that PLOS defines the minimal data set as the underlying data used to reach the conclusions in the manuscript and any additional data required to replicate the reported study findings in their entirety. We apologize for the oversight in not specifying where the minimal data set can be found.

We have updated the data availability statement to include the location of the minimal data set. The minimal data set can now be found at Supporting Information. Furthermore, it should be noted that the studies included in our research have been cited, and readers can easily access the corresponding data within these studies. We have ensured that the data set is fully available and can be accessed by readers to replicate our findings.

Thank you for bringing this to our attention and please let us know if you have any further concerns or suggestions.

4. PLOS requires an ORCID iD for the corresponding author in Editorial Manager on papers submitted after December 6th, 2016. Please ensure that you have an ORCID iD and that it is validated in Editorial Manager. To do this, go to ‘Update my Information’ (in the upper left-hand corner of the main menu), and click on the Fetch/Validate link next to the ORCID field. This will take you to the ORCID site and allow you to create a new iD or authenticate a pre-existing iD in Editorial Manager. Please see the following video for instructions on linking an ORCID iD to your Editorial Manager account: <https://www.youtube.com/watch?v=_xcclfuvtxQ>

Response: Thank you for bringing to my attention the requirement for the corresponding author to have an ORCID iD in Editorial Manager for papers submitted.
 I have created a new ORCID iD and have validated it in Editorial Manager as per the instructions provided. To link my ORCID iD to my Editorial Manager account, I followed the steps outlined in the video tutorial provided and successfully completed the process.
Thank you for your assistance in ensuring that my submission meets the necessary requirements.

5.Please include captions for your Supporting Information files at the end of your manuscript, and update any in-text citations to match accordingly. Please see our Supporting Information guidelines for more information: <http://journals.plos.org/plosone/s/supporting-information>.

Response: Thank you for your valuable feedback. I have carefully reviewed your suggestion and have made the necessary changes by including captions for all the Supporting Information files at the end of the manuscript.

I appreciate your guidance and have followed the guidelines provided in the Supporting Information guidelines for PLOS ONE. Please let me know if there are any further changes or revisions that you would like me to make.

**Response to Reviewer #1：**

1- Usually subjects with abnormal lipid profile receive medications which may or may not affect the occurrence of final outcome which is gastric cancer. What measures or strategies authors used to overcome such bias?

Response: We appreciate your concern regarding potential biases introduced by the use of lipid-lowering medications among study participants.

To address the potential bias, we conducted subgroup analyses and a meta-regression to investigate the impact of lipid-lowering medication usage, which are presented in Table 1. Additionally, we performed sensitivity analyses. We believe these measures effectively controlled for the influence of lipid-lowering medication usage on the study findings, ensuring the reliability and validity of our research.

2- In some of included studies the RR was related to risk highest levels of measured lipid (for example TC) compare to lowest levels (Ahn et al 2009), while in some other studies only the risk of levels was evaluated. How the authors adjusted this?

Response: Thank you for your feedback. In response to the concern about the correction of blood lipid levels in our study, we would like to clarify that we carefully evaluated the methods and results of each study to determine appropriate adjustments.
When extracting effect sizes, we obtained hazard ratios (HR) or relative risks (RR) with the most comprehensive adjustment for confounding factors and their corresponding 95% confidence intervals. In addition, we performed subgroup analyses for confounding factors that were adjusted in the included studies.
We believe that our measures adequately addressed potential confounding effects and provided a more accurate estimate of the association between blood lipid levels and the risk of the outcome of interest.

3- there should be a table presenting the characteristics of studies, including the year of publication, number of subjects in each group, duration, ….

Response: Thank you for your feedback. In response to the suggestion regarding the presentation of the characteristics of the included studies, we have created a separate document named "S2 Table" to provide a comprehensive overview of the year of publication, number of subjects in each group, duration, and other relevant information for each study.

We apologize for any confusion caused by our initial placement of this information in the supplementary materials as Table S2. We hope that this revised approach will make it easier for readers to access and evaluate the characteristics of the included studies.

4- there are some grammatical errors. The manuscript needs to be revised grammatically.

Response: Thank you for reviewing our manuscript and for providing feedback on the grammatical errors. We apologize for any inconvenience caused by the grammatical errors in our initial submission.

We have thoroughly revised the manuscript to correct all of the grammatical errors. We have carefully reviewed and edited the text to ensure that it meets high standards of grammar and style. Additionally, we had our manuscript reviewed by a professional editor to ensure its quality.

Once again, we thank you for bringing this to our attention and we appreciate your valuable feedback. Please let us know if you have any further suggestions or comments.

**Response to Reviewer #2：**

1- There should be no references in the abstract.

Response: Thank you for your feedback. We appreciate your suggestion regarding the references in the abstract. We have carefully reviewed our manuscript and removed all references from the abstract to comply with the journal's guidelines.

2- GC has not been defined on its appearance in the introduction.

Response: Thank you for your feedback. We have carefully reviewed our manuscript and have made the necessary revisions to define the term GC in the introduction. We have highlighted the revised text in yellow for your convenience.

3- I believe a Table containing the summary findings of each included studies should be included in the main text.

Response: Thank you for your feedback. We appreciate your suggestion regarding the inclusion of a summary table for the findings of each included study in the main text.
We would like to clarify that we have already addressed this concern in our previous correspondence. We have created a separate document named "S2 Table" to provide a comprehensive overview of the characteristics and findings of each included study. This table will be included in the resubmission of our manuscript.
We apologize for any confusion caused by the initial placement of this information in Table-S2 in our initial submission. We hope that the revised approach will make it easier for readers to access and evaluate the findings of the included studies.

4- I could not find the supplemental data so I was not able to assess the manuscript fully.

Response: Thank you for bringing to our attention the issue regarding the supplemental data. We apologize for any inconvenience caused by the inability to locate the data in our initial submission.
We have carefully reviewed and revised the supplemental data according to PLOS ONE guidelines and have reorganized it to make it easily accessible in the revised submission. We have also provided clear instructions for locating the supplemental data in the cover letter.
We hope that this revised approach will enable you to access and assess the supplemental data fully. We appreciate your valuable feedback, and we remain committed to ensuring that all components of our manuscript are presented accurately and transparently.
