# Supplementary material for: Association between blood lipid levels and risk of gastric cancer: A systematic review and meta-analysis
Source: PLoS One. 2023 Jul 7;18(7):e0288111. doi: 10.1371/journal.pone.0288111 (PMC10328306; doi:10.1371/journal.pone.0288111)
Supplement: Attachment — Submitted filename: Response to Reviewers.docx [file pone.0288111.s007.docx]

Dear Academic Editor and Reviewers:

Thank you for your prompt response and valuable feedback on our manuscript titled **Association between blood lipid levels and risk of gastric cancer: A systematic review and meta-analysis.** We sincerely appreciate the time and effort you have dedicated to reviewing our work and providing constructive comments for improvement. We have carefully considered your suggestions and have made the necessary revisions accordingly. The following represents our response to your valuable feedback.

**Response to Academic Editor:**

1.Please re-upload PRISMA flowchart in accordance with the attached file. Moreover, consider minor English language editing in whole manuscript.

**Response:** Thank you for your valuable feedback. We appreciate your suggestion. We have made the necessary changes and have re-uploaded the PRISMA flowchart in accordance with the attached file. Furthermore, we have carefully reviewed the entire manuscript and have made minor English language edits where necessary. Thank you for bringing this to our attention, and we believe the revised version now meets the required standards.

2.Please review your reference list to ensure that it is complete and correct. If you have cited papers that have been retracted, please include the rationale for doing so in the manuscript text, or remove these references and replace them with relevant current references. Any changes to the reference list should be mentioned in the rebuttal letter that accompanies your revised manuscript. If you need to cite a retracted article, indicate the article’s retracted status in the References list and also include a citation and full reference for the retraction notice.

**Response:** Thank you for your valuable feedback and guidance on our manuscript. We have thoroughly reviewed the reference list and can confirm that it is complete and correct. We have carefully checked each reference and have not found any errors or retractions in the cited papers.

Based on our thorough examination, we are confident that our reference list accurately represents the relevant and current literature in our field of study. We have taken great care to ensure the integrity of our citations, and we believe that the references provided in our manuscript are reliable and appropriate for supporting our research.

We appreciate your diligence in highlighting the importance of maintaining an accurate and up-to-date reference list, and we are grateful for the opportunity to address this aspect of our manuscript. Should you have any further concerns or suggestions, please do not hesitate to let us know.

Thank you once again for your thorough review, and we look forward to your further evaluation of our revised manuscript.

**Response to Reviewer #1：**

**Response:** Thank you for reviewing our manuscript and for your previous comments. We appreciate your acknowledgement that the previous comments have been addressed by the authors.

We have carefully reviewed the manuscript once again, focusing specifically on spelling and minor grammatical errors. We have made the necessary corrections to ensure the accuracy and clarity of the text. Additionally, we have conducted a thorough proofreading to improve the overall language quality.

We sincerely appreciate your attention to detail and your efforts in helping us enhance the manuscript's readability. Your input has been invaluable in improving the overall quality of our work.

If you have any further comments or suggestions, please do not hesitate to let us know.

**Response to Reviewer #2：**

**I thank the authors for their detailed revision. All my comments have been addressed and I believe the manuscript can be considered for potential publication.**

Response: Thank you for your thorough review of our revised manuscript. We sincerely appreciate your time and effort in providing constructive feedback and suggestions. We are pleased to hear that you acknowledge our efforts in addressing all of your comments and that you believe the manuscript is now suitable for potential publication.

Your positive evaluation of our revised manuscript is encouraging and greatly appreciated. We are grateful for the opportunity to have our work considered for publication in PLOS ONE. We would like to express our gratitude for your valuable input, which has undoubtedly contributed to the improvement of our research.

Should there be any further steps or requirements in the publication process, please kindly let us know, and we will promptly address them.

Once again, thank you for your time and support. We look forward to the possibility of our manuscript being published in PLOS ONE.
