# Supplementary material for: Association between blood lipid levels and risk of gastric cancer: A systematic review and meta-analysis
Source: PLoS One. 2023 Jul 7;18(7):e0288111. doi: 10.1371/journal.pone.0288111 (PMC10328306; doi:10.1371/journal.pone.0288111)
Supplement: Attachment — Submitted filename: Response to Reviewers.docx [file pone.0288111.s008.docx]

Dear Academic Editor,

I would like to express my sincere gratitude to the academic editor for their valuable assistance and suggestions. I have made the necessary revisions based on your feedback and have provided my response below.

Please insert the correct format of the Fig-1 that observed in revised manuscript with track changes file.

Response: Thank you for your feedback on my PRISMA flowchart. I have thoroughly reviewed and revised the flowchart, taking into consideration the format used in previously published studies in PLOS ONE. The flowchart has now been modified to align with the appropriate format. I appreciate your guidance in ensuring the accuracy and adherence to the journal's standards. Please let me know if there are any further revisions or modifications you would like me to address.
